# A new and effective method to induce infection of *Phyllachora maydis* into corn for tar spot studies in controlled environments

**DOI:** 10.1186/s13007-023-01052-8

**Published:** 2023-08-11

**Authors:** José E. Solórzano, Shea E. Issendorf, Milton T. Drott, Jill C. Check, Emily M. Roggenkamp, C. D. Cruz, Nathan M. Kleczewski, Carlos C. Gongóra-Canul, Dean K. Malvick

**Affiliations:** 1 Department of Plant Pathology, University of Minnesota, St. Paul, MN 55108 U.S.A.; 2 United States Department of Agriculture-Agricultural Research Service, Cereal Disease Laboratory, St. Paul, MN 55108 U.S.A.; 3 Department of Plant, Soil and Microbial Sciences, Michigan State University, East Lansing, MI 48824 U.S.A.; 4 Department of Botany and Plant Pathology, Purdue University, West Lafayette, IN 47907 U.S.A.; 5 GROWMARK Agronomy Services, Bloomington, IL 61702 U.S.A.; 6 Tecnológico Nacional de México, Instituto Tecnológico de Conkal, Conkal, YU 97345 Mexico

**Keywords:** Disease, Biotroph, Black spot, Fungus, Inoculation, Maize, Method, Replicable

## Abstract

**Background:**

Tar spot of corn is a significant and spreading disease in the continental U.S. and Canada caused by the obligate biotrophic fungus *Phyllachora maydis.* As of 2023, tar spot had been reported in 18 U.S. states and one Canadian Province. The symptoms of tar spot include chlorotic flecking followed by the formation of black stromata where conidia and ascospores are produced. Advancements in research and management for tar spot have been limited by a need for a reliable method to inoculate plants to enable the study of the disease. The goal of this study was to develop a reliable method to induce tar spot in controlled conditions.

**Results:**

We induced infection of corn by *P. maydis* in 100% of inoculated plants with a new inoculation method. This method includes the use of vacuum-collection tools to extract ascospores from field-infected corn leaves, application of spores to leaves, and induction of the disease in the dark at high humidity and moderate temperatures. Infection and disease development were consistently achieved in four independent experiments on different corn hybrids and under different environmental conditions in a greenhouse and growth chamber. Disease induction was impacted by the source and storage conditions of spores, as tar spot was not induced with ascospores from leaves stored dry at 25 ºC for 5 months but was induced using ascospores from infected leaves stored at -20 ºC for 5 months. The time from inoculation to stromata formation was 10 to 12 days and ascospores were present 19 days after inoculation throughout our experiments. In addition to providing techniques that enable in-vitro experimentation, our research also provides fundamental insights into the conditions that favor tar spot epidemics.

**Conclusions:**

We developed a method to reliably inoculate corn with *P. maydis*. The method was validated by multiple independent experiments in which infection was induced in 100% of the plants, demonstrating its consistency in controlled conditions. This new method facilitates research on tar spot and provides opportunities to study the biology of *P. maydis*, the epidemiology of tar spot, and for identifying host resistance.

**Supplementary Information:**

The online version contains supplementary material available at 10.1186/s13007-023-01052-8.

## Background

Tar spot of corn (*Zea mays* L.) is caused by the fungus *Phyllachora maydis* in North, Central, and South America, and the Caribbean. This crop disease was first confirmed in the continental United States (U.S.) in 2015 [1]. As of July 2023, tar spot had been documented in 18 states and Ontario, Canada, and significant grain-yield losses were reported across corn-producing regions over multiple years [2–4]. The recent emergence of the disease and the dearth of information have made the disease a significant issue, prompting the need for studies to understand the interactions of corn with *P. maydis* and develop tar spot management strategies. However, research has been limited by an inability to reliably induce tar spot under experimental conditions.

*P. maydis* is a member of the order Phyllachorales in the family Phyllachoraceae. Fungi in this order are thought to be obligate biotrophs and require a living host for growth and reproduction [5]. Records of the genus *Phyllachora* indicate these fungi occur globally except in the Arctic and Antarctica, are obligate biotrophs, and colonize monocots and dicots [6]. In general, the presence of *Phyllachora* species in their host plant can be observed when black stromata form. These structures typically contain conidia-bearing pycnidia and/or ascospores-bearing perithecia [7]. While conidia are produced in large numbers by *Phyllachora* species, there is ambiguity about their functionality because they are not known to germinate or cause infection. Furthermore, ascospores are the only known source of infection for *P. maydis* and other *Phyllachora* species.

The proposed tar spot disease cycle begins with the dispersal of ascospores onto corn leaves under favorable environmental conditions [8]. Early chlorotic symptoms then can develop followed by the formation of black stromata that are raised, embedded in the tissue, and may be surrounded by necrosis [9]. The stromata often extrude masses of ascospores [9] that allow multiple cycles of infection during the growing season [8]. Ascospores overwinter within the stromata on infected foliage and can be dispersed in the following growing season under favorable environmental conditions [10].

Tar spot signs have been observed across vegetative (V) and reproductive (R) stages of corn [11, 12]. The disease is diagnosed on infected plants when signs (stromata) of *P. maydis* have developed in the tissue. To date, the factors that result in successful infection of *P. maydis* have yet to be fully confirmed [12]; however, most available information suggests that temperatures from 16 to 23 °C coupled with wet and humid conditions favor infection and disease establishment [13].

Efforts to manage tar spot in the U.S. have mainly relied on fungicide applications [14]; however, the most effective timing of these applications has been difficult to establish because the incubation time of the disease and the factors that influence disease progression under different environmental conditions are poorly understood. Efforts to breed for tar spot resistance have relied on natural infection in the field and have been hampered by high levels of variability in disease prevalence, sometimes confounding the role of host genetics in disease mitigation [12]. However, field studies of exotic corn germplasm have identified several lines with relatively low susceptibility to tar spot [12]. Rapid advancements in these areas and for integrated management of tar spot will be facilitated by an effective and reliable method to induce tar spot in controlled conditions.

In this study, we developed a method to inoculate corn plants with *P. maydis* to induce tar spot based on an enhanced understanding of the tar spot pathosystem. It provides a consistent and efficient method to infect corn with *P. maydis*. The method will enable efforts to identify tar spot resistance in corn germplasm, evaluate tar spot management tactics, and conduct research into the biology of *P. maydis* and its interactions with plant hosts.

## Results

### Preliminary experiments and evaluation of existing methodologies

We assessed the replicability of three published protocols [10, 15] to induce tar spot with two corn hybrids (H1 and H3; Table 1). Hybrid H3 was known to be susceptible to *P. maydis* from field studies in Minnesota, and hybrid H1 had unknown resistance/susceptibility to *P. maydis*. Several previously published protocols were tested: (i) inoculating and covering inoculated plants with bags, conducted once [15]; (ii) inoculation of detached leaves, conducted twice [15]; and (iii) inoculation of plants with a hand atomizer, conducted four times [10]. Despite all protocol steps being followed as reported, evaluated methodologies led to no induction of tar spot.

**Table 1:** Corn hybrids (*Zea mays* L.) used for inoculation experiments

Hybrid code	Common name- Hybrid	Source company
H1	Corn NK9653-5222‡	Syngenta® ^a^
H2	Corn NK9610-5122-EZ1‡	Syngenta® ^a^
H3	Corn GC-103-58 RSS †	Gold Country Seed® ^b^
H4	Sweet corn 5456T.54‡	Johnny’s Selected Seeds ^c^

† Hybrid known to be susceptible to tar spot from field observations

‡ Unknown susceptibility to tar spot

a 1330 Lagoon Ave, Minneapolis, MN 55408, U.S.A.

b 4777 Shady Oak Rd, Hopkins, MN 55343, U.S.A.

c 955 Benton Ave., Winslow, ME 04901, U.S.A.

Conidia alone did not induce tar spot when used as inoculum. We manually collected extruded orange to red masses from stromata 18 days after field-infected plants were moved to a greenhouse and confirmed the presence of conidia through microscopy. Using the collected conidia, we inoculated 16 plants of hybrid H3 at the V3 growth stage (8 in a greenhouse [19 to 22 °C] and 8 in a mist chamber [20 to 25 °C, 48 h at 100% RH with humidifying cycles of 10 min., every 120 min] + greenhouse [19 to 22 °C]). The control group contained 16 non-inoculated plants of the same growth stage that were exposed to the same conditions as the inoculated plants. The plants were observed for 30 days after inoculation (dai); however, tar spot signs did not form despite conidia being abundant in the inoculum (10^7^ conidia/ml) (see methods). Disease signs or symptoms also did not appear in non-inoculated plants.

It was determined that field-infected green leaves containing *P. maydis* stromata can be used directly to induce tar spot, but results were not consistent. With a proximity inoculation test, infected leaf pieces (3 cm wide) were fixed to healthy leaves (see methods) and inconsistent induction of tar spot was achieved in a growth chamber (19 °C, 85 to 100% RH, 8-h photoperiod). The first test resulted in tar spot induction on the single plant evaluated, but only 2 of 10 plants (20%) in a subsequent test. In the first test, mild chlorotic symptoms appeared 4 dai and multiple stromata formed 8 dai. Stromata continued to appear over the following 30 days in the same leaf area, and stromata did not develop on non-inoculated parts of the leaves. However, in the second test, only one stroma of *P. maydis* appeared on each infected plant, and chlorosis was not observed before the stromata formed. Based on the inconsistent success in initiating tar spot with these methods, conidia alone and the direct use of field-infected green leaves were not considered reliable inocula to initiate tar spot.

### Development of the new methodology

**Fig. 1:**
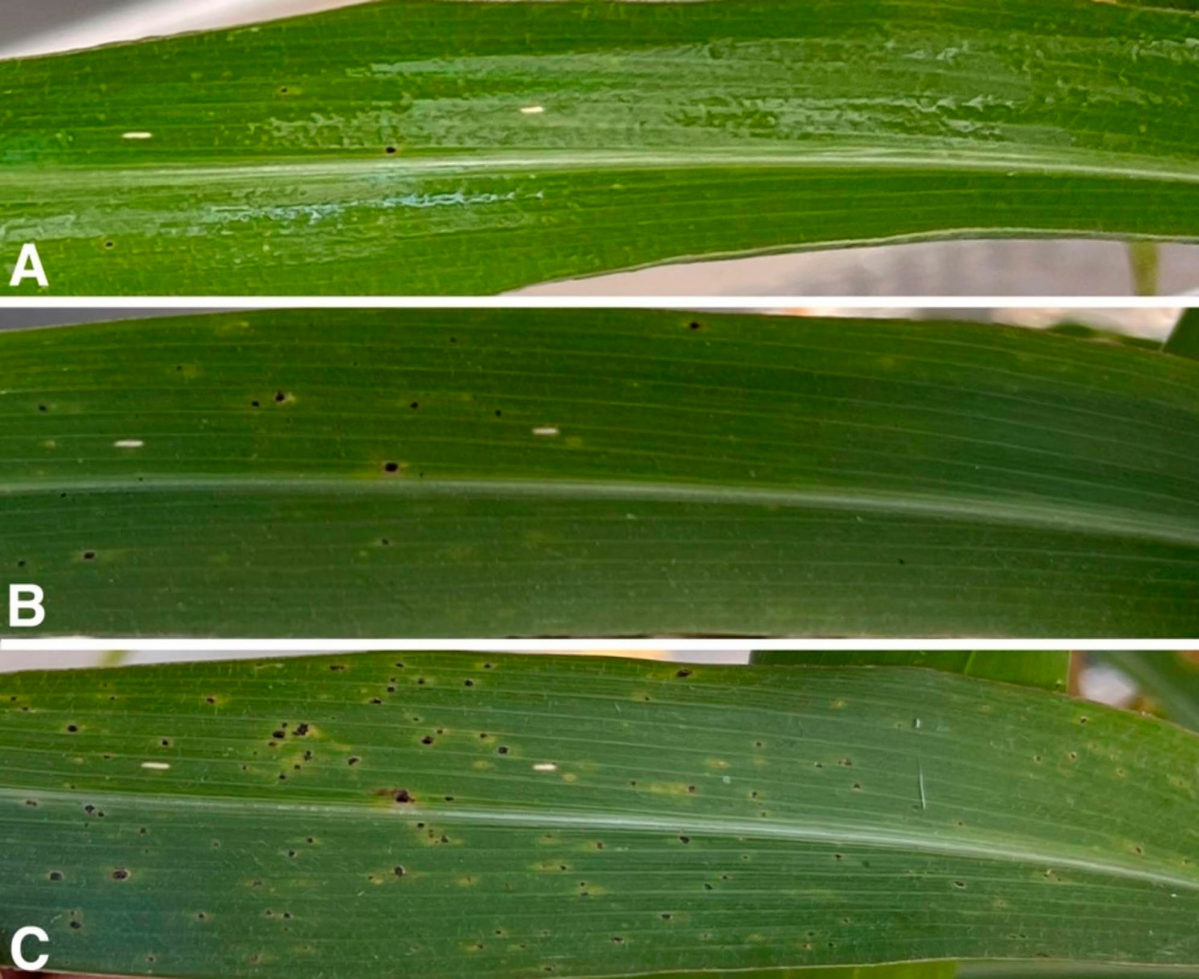
Progression of tar spot symptoms and signs on a single corn (*Zea mays* L.) leaf from a field-infected plant incubated under greenhouse conditions (Supplementary Table 1). (**A**). 1 day after collection from the field. (**B**). 7 days after collection from the field. (**C**). 13 days after collection from the field

**Fig. 2:**
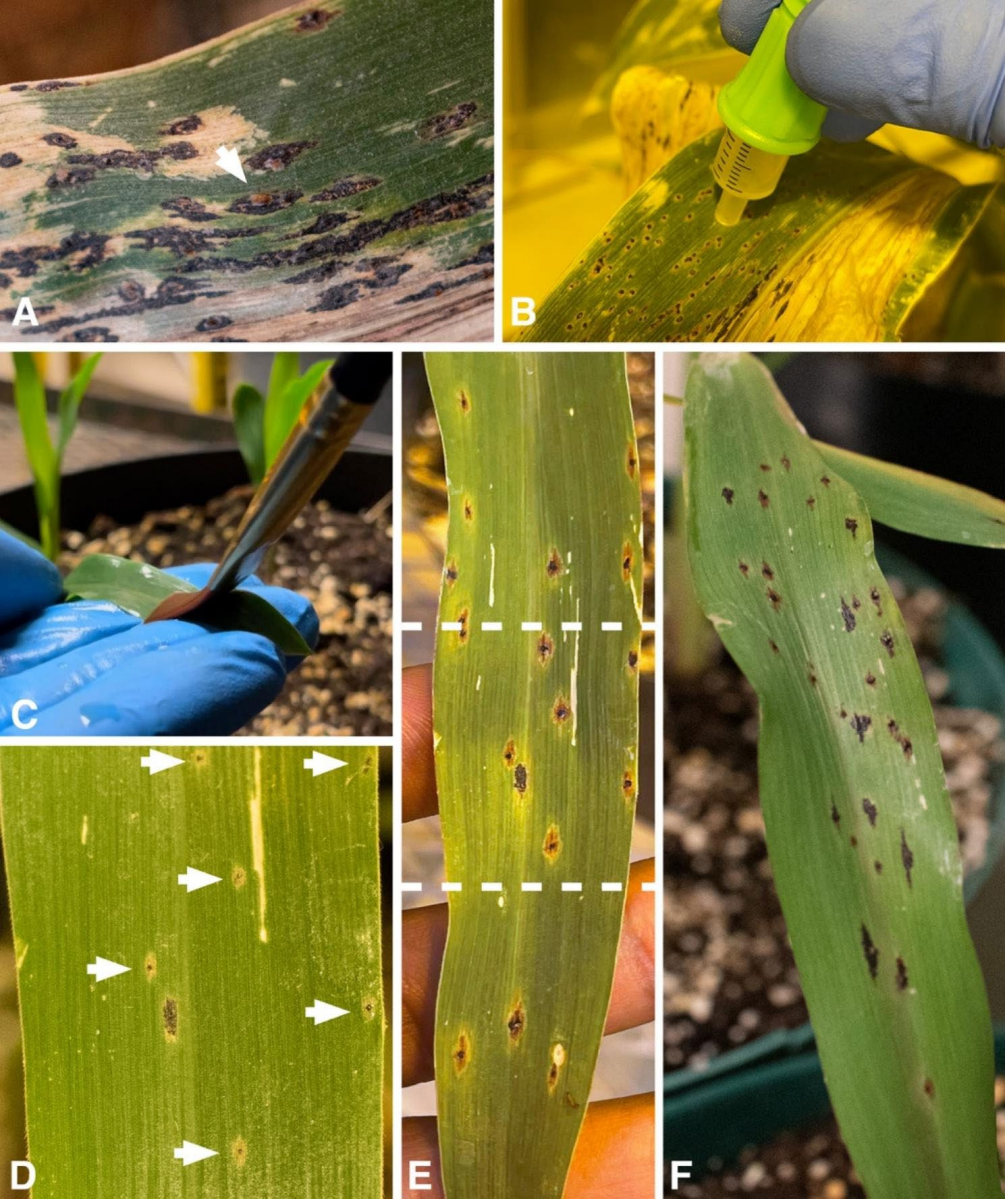
Representation of the induction of tar spot using the new inoculation method from spore collection to stromata development. (**A**). Stromata of *Phyllachora maydis* with extruded spores (note orange mass marked with an arrow) before collection. (**B**). Collection of spores from field-infected plants using the syringe tip attached to the vacuum. (**C**). Inoculation of corn leaves by application of spore suspension using a brush. (**D**). Stromata arising from flecked chlorotic lesions 12 dai (see white arrows). (**E**). Morphology of stromata 20 dai, where the area between the dashed lines is the area shown in (**D**). (**F**). Morphology of stromata 18 dai

To ensure the availability of infectious spores of *P. maydis*, field-infected plants were collected from a field in southern Minnesota and spores were collected from them using a vacuum collection device or syringe tip (Supplementary Fig. 1). The plants were moved from the field to a greenhouse (see methods and Supplementary Table 1), where symptoms of infection and stromata of *P. maydis* continued to develop (Fig. 1). The continued progression of tar spot served as evidence that greenhouse conditions were favorable for disease development. Moreover, 7 days after field-infected plants were introduced to the greenhouse, the stromata started extruding spores that remained attached to the stromata for > 10 days or until collection (Figs. 1 and 2A). Since conidia were not found to be infectious (see above), we targeted collection of ascospores from the stromata using the vacuum collection device by rubbing the collector against the surface of the stromata (Fig. 2B). During this process, ascospores and conidia were collected. Ascospores germinated within 30 min (Supplementary Fig. 2), but conidia did not germinate within 24 h when the mixed spore suspensions were suspended in 0.01% Tween 20 at 24 ± 1 °C. While only ascospores are known to infect corn, a mixture of ascospores and conidia was further used for inoculation due to the time required and the difficulty to separate the spore types.

**Fig. 3:**
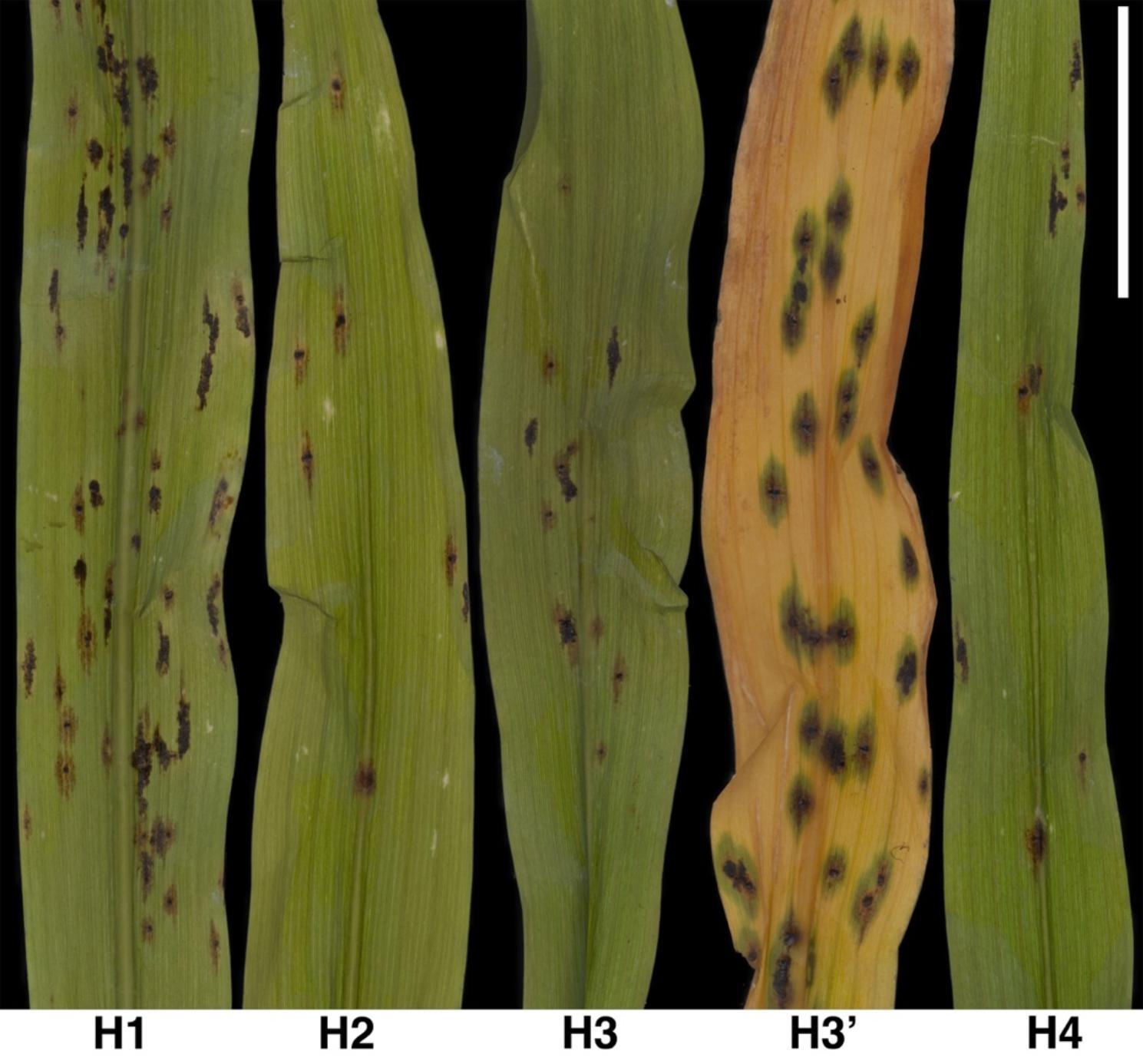
Phenotypic representation of the development of *Phyllachora maydis* 19 dai (7 days after the first stroma formed) in four corn (*Zea mays* L.) hybrids (H1 to H4; Table 1) (Table 5 and Fig. 4). All hybrids were susceptible to *P. maydis*, and the number of stromata occasionally varied among them (Table 5 and Fig. 4). Hybrid **H3’** shows a senescent corn leaf as occasionally seen with stromata surrounded by green halos. Bar = 2 cm

To assess infection capacity, ascospores, and conidia were collected from two field-infected plants 27 days after the plants were moved to the greenhouse (experiment 1). The spore mixture was applied to the leaves of 14 hybrid H3 plants at the V2 growth stage with a flat paint brush within 1 h after spore collection (see methods). The control group contained 14 non-inoculated plants (experiment 1, Table 2). Following inoculation, the plants were placed in a mist chamber where a humidifier was set to cycles of 10 min every 120 min to maintain continuous 100% RH and leaf wetness (Table 3). After 20 h in the mist chamber, 10 inoculated and 10 non-inoculated plants were moved to a greenhouse (Tables 2 and 4), and 4 inoculated and 4 non-inoculated plants were moved to a growth chamber (Tables 2 and 4). Stromata appeared on leaves 11 to 12 dai in all inoculated plants in the greenhouse and growth chamber. Occasionally, chlorotic flecking was observed 1 to 2 days before the formation of small, brown stromata (Figs. 2D to F and 3). Chlorotic symptoms alone, however, were not indicative of infection since they were not always present before stromata formed. Green halos were also occasionally observed around the stromata on senescing leaves (Fig. 3H3’). It was noted that leaves extending horizontally during inoculation usually developed more stromata compared to those that extended vertically (e.g., Figs. 2D to F and 3H1). All stromata that resulted from these inoculations resembled those observed on naturally infected plants (Figs. 2 and 3). Stromata did not develop on non-inoculated leaves or on any non-inoculated plants.

**Table 2:** Experimental arrangement for testing of the new method

Experiment	Location	Group	Spore source	Plants ‡
1	Greenhouse	New method	Infected plants	10
		Control	*NA*	10
	Growth chamber	New method	Infected plants	4
		Control	*NA*	4
2	Greenhouse	New method	Infected plants	51
		Control †	Dried leaves	9
	Growth chamber	New method	Infected plants	21
		Control †	Dried leaves	4
3	Greenhouse	New method	Infected plants	42
		Control †	Dried leaves	21
4	Greenhouse	New method	Green leaves	27
		New method	Dried leaves	28

*NA* Non-inoculated plants

† Plants were inoculated using a hand atomizer

‡ Number of plants evaluated

**Table 3:** Temperature and percentage relative humidity for induction of tar spot in a mist chamber

	Range	% RH	Temperature ºC
*60 min before inoculation* †			
	Minimum	48	20
	Mean	90	21
	Maximum	100	28
*For 20 h* ‡			
	Minimum	100	19
	Mean	100	20
	Maximum	100	21

Data were collected in dark (0 µmol/m^2^/s) while using the new inoculation method

† The humidifier was set to increase % RH to 100% before inoculation

‡ Once all the inoculated plants were in the mist chamber the humidifier ran cycles of 10 min every 120 min to maintain continuous 100% RH for 20 h

**Table 4:** Temperature and percentage relative humidity for the growth and development of *Phyllachora maydis* on corn in greenhouse and growth chamber experiments

Experiment	Location	Range	% RH §	Temperature ºC §	Light intensity µmol/m^2^/s
1	Greenhouse	Minimum	32	22	163
		Mean	45	23	230
		Maximum	62	24	265
	Growth chamber	Minimum	*NA*	*NA*	211
		Mean	*NA*	*NA*	240
		Maximum	*NA*	*NA*	258
2	Greenhouse	Minimum	19	22	137
		Mean	35	23	199
		Maximum	54	25	230
	Growth chamber	Minimum	29	19	131
		Mean	40	20	216
		Maximum	48	20	320
3	Greenhouse	Minimum	30	23	163
		Mean	37	23	273
		Maximum	54	25	352
4	Greenhouse	Minimum	29	20	97
		Mean	45	21	267
		Maximum	54	24	536

Data were collected while using the new inoculation method

§ Values were collected for 12 days based on the average time to first stroma (Table 5)

*NA* During experiment 1, temperature and % RH were not recorded in the growth chamber due to technical difficulties. However, the growth chamber was set to 19 °C, 85 to 100% RH, and 8-h photoperiod

**Fig. 4:**
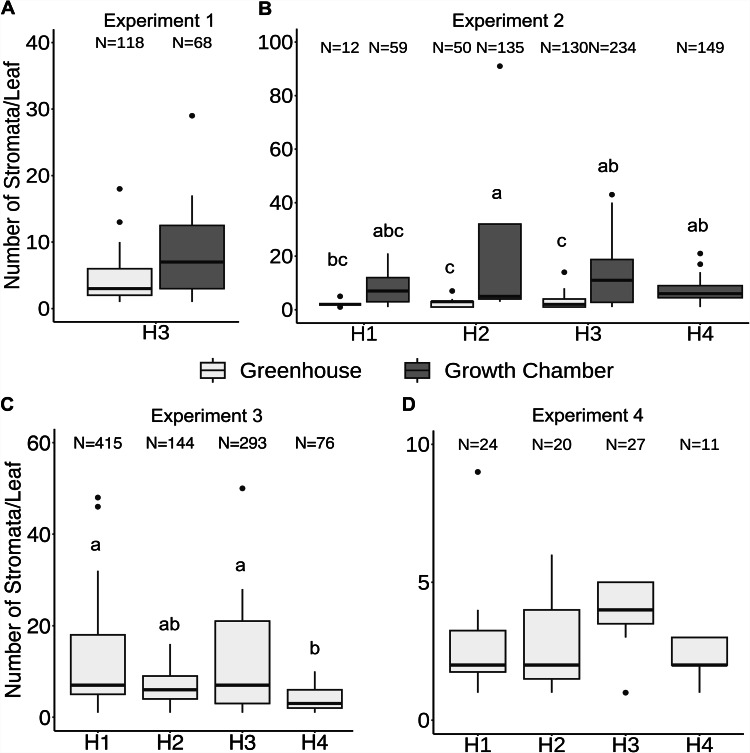
Development of *Phyllachora maydis* stromata on corn leaves in four corn (*Zea mays* L.) hybrids (H1 to H4; Table 1) in growth chamber or greenhouse conditions after using the new inoculation method. Stromata of *P. maydis* developed on 100% of the inoculated plants in all experiments when spores were collected from field-infected plants located in the greenhouse (experiments 1 to 3) or field-infected green leaves stored at -20 ºC for 5 months (experiment 4). Note that the scale on the y-axis varies between experiments. **A**. Number of *P. maydis* stromata per leaf 26 dai in a growth chamber and greenhouse. **B**. Number of *P. maydis* stromata 19 dai in a growth chamber and greenhouse. **C** and **D**. Number of *P. maydis* stromata per leaf 19 dai in a greenhouse. “N” equals the total number of stromata of *P. maydis* counted from each hybrid in the given experiment. Different letters in **B** and **C** (experiments 2 and 4) denote statistical differences as determined by Tukey’s Honestly Significant Difference (*P* < 0.05). In **A** and **D** (experiments 1 and 4), the development of *P. maydis* was not statistically different (*P* > 0.05)

To allow the growth and development of *P. maydis*, stromata were counted 26 dai but leaves became difficult to phenotype in the greenhouse due to natural senescence. In contrast, infected leaves of plants grown under growth chamber conditions stayed green longer which facilitated phenotyping. In experiment 1, no significant differences (*P* = 0.21) in disease severity (number of stromata per leaf) were observed between greenhouse and growth chamber conditions or between the inoculated leaves of each hybrid (*P* = 0.80) (Fig. 4A; Supplementary Table 2).

### Validation of the new inoculation method

To assess replicability of the new inoculation method and consistency of tar spot induction, two independent experiments (experiments 2 and 3) were conducted using spores collected at different time points from field-infected plants. These experiments included a larger number of plants and four different corn hybrids (H1 to H4; Tables 1 to 5).

For experiment 2, spores were collected from tar spot stroma from two field-infected plants 54 days after being moved to a greenhouse and were used for inoculation within 1 h after collection. The control group consisted of 9 plants in the greenhouse and 4 plants in the growth chamber, which were inoculated using a hand atomizer containing spores from dried leaves (Table 2). After inoculation and 20 h in the mist chamber, 51 plants were moved to the greenhouse (Tables 2 and 4) and 21 plants were moved to a growth chamber (Tables 2 and 4). With the new method, tar spot was induced in 100% of the inoculated plants but not in the control group (Table 5). Stromata first appeared on leaves 11 to 12 dai, a result that was similar to experiment 1. In contrast with experiment 1 where leaves senesced 26 dai, phenotyping was facilitated by collecting infected leaves 19 dai before senescence. Disease severity was significantly different among the four hybrids under greenhouse and growth chamber conditions (*P* = 0.00) but not between the inoculated leaves of each hybrid (*P* = 0.47). Disease severity was significantly different (*P* < 0.05) between corn hybrids in growth chamber and greenhouse conditions, e.g., H2 vs. H1, H3 vs. H2, H4 vs. H2, H2 vs. H3, and H4 vs. H3. (Supplementary Table 3).

To further assess the replicability of the new method, a third experiment (experiment 3) was conducted by collecting spores from the two field-infected plants 3 days after spores were collected in experiment 2. Similar to experiment 2, spores were used within 1 h after collection and four hybrids (H1 to H4; Table 1) were evaluated in greenhouse conditions with 41 plants. A control group of 21 plants was inoculated as in experiment 2 using a hand atomizer with *P. maydis* spores from dried leaves. Tar spot stromata developed in 100% of the inoculated plants and the stromata first appeared 11 to 12 dai (Table 5; Fig. 4C), which is consistent with experiments 1 and 2. Tar spot was not induced in the control group. Phenotyping was also facilitated by collecting green leaves 19 dai. Statistical differences in disease severity were observed between hybrids (*P* = 0.00) in particular between H4 and H3 (*P* = 0.03) and between H1 and H4 (*P* = 0.00), but disease severity was not significantly different between the inoculated leaves of each hybrid (*P* = 0.15) (Supplementary Table 4).

### Validation of the method using detached field-infected leaves as a source of spores

A fourth independent experiment (experiment 4) was conducted to determine if spores from green leaves stored at -20 °C for 5 months could be used to induce tar spot using the new inoculation method. Spores were collected with the vacuum collection device (Supplementary Fig. 1) from stroma on the leaves that had been frozen (the same leaves used for the proximity test) and from dried leaves that were stored at 24 ± 1 °C for 5 months. The spores were used within 2 h after collection to inoculate four hybrids (H1 to H4; Table 1). No stromata were observed on plants inoculated with spores from dried leaves (Table 5). Tar spot was induced in 100% of the 27 plants inoculated with spores from green leaves (Fig. 4D). The stromata appeared 10 dai on two plants of hybrid H3 and 11 dai in the remaining 25 inoculated plants. Disease severity was not significantly different between hybrids (*P* = 0.48; Supplementary Table 5) in this experiment. This result suggests that spore source and storage conditions are determinant factors for the success of the inoculation method since tar spot was not induced using spores from dried leaves stored at 24 ± 1 ºC.

**Table 5:** Experimental location, time to first stroma, infection success, and counts of *Phyllachora maydis* stromata

Experiment	Location	Average time to first stroma	Plants †, ‡	Total infected leaves	Total stromata *	
1	Greenhouse	12	10	25	118	bcd
	Growth chamber	12	4	7	68	abc
2	Greenhouse	12	51	70	192	d
	Growth chamber	12	21	47	428	a
3	Greenhouse	12	42	99	928	ab
4	Greenhouse	11	27	27	82	cd

Data collected after inoculation using the new method

† 3 to 4 leaves were inoculated per plant in experiments 1 to 3 and 1 leaf per plant was inoculated in experiment 4

‡100% of inoculated plants developed stromata. In experiment 4, tar spot was not induced in plants inoculated with spores from dried leaves

*Different letters represent statistical differences among experimental group conditions (Tukey’s Honestly Significant Difference, *P* < 0.05). Control groups are not included since stromata did not form in them

In these four experiments (Table 5; Fig. 4), the replicability and consistency of the new inoculation method were confirmed by successfully inducing tar spot under different greenhouse and growth chamber conditions using different sources of inoculum and across four corn hybrids (H1 to H4; Figs. 2 and 3, and 4; Table 5). Disease severity between inoculated leaves was significantly different among experiments (Supplementary Table 6; *P* = 0.00) but not within each experiment (Supplementary Tables 2 to 5; *P* > 0.05). In general, disease severity was significantly different among all experiments (*P* = 0.00), but no statistical differences (*P* > 0.05) were observed between growth chamber experiments (1 and 2) and greenhouse experiments (1 and 2, 3 and 1, 4 and 1, and 4 and 2) or between some greenhouse vs. growth chamber experiments (1 and 1, 3 and 1, 4 and 1, and 3 and 2, respectively).

## Discussion

The need for a reliable inoculation method has limited the study of tar spot. This includes studies of factors influencing host-pathogen interactions and the ability to identify resistance traits for genetic improvement of corn, which is urgently needed for effective management. Additionally, the non-replicability of methods to induce tar spot and the lack of information about the disease have impeded the completion of Koch’s postulates for the first reports of the disease since 2015 [1] to 2023 [16] in the U.S. and Canada. In this study, we developed an effective and consistent method to induce tar spot of corn in controlled conditions. The new inoculation method is based on observations of natural tar spot infections to inform the conditions needed to achieve consistent and replicable tar spot infection in greenhouse and growth chamber conditions.

Several reports have reported methods for the induction of tar spot in controlled conditions [10, 15, 17, 18]. However, in our evaluation of three of those methods [10, 15] we were unable to replicate the reported results and obtained no infections with the prescribed methodologies. Based on the results obtained using the new method, we speculate that different factors may have hindered our successful induction of tar spot using the previously described methods, e.g., the storage conditions and storage time of infected tissue, the viability of infectious spores in the tissue, or temperature, light, and humidity conditions required for the establishment of the disease. Therefore, to induce tar spot, this study evaluated conditions important for disease development, including spore source, tissue storage, and ranges of humidity and temperature. The new inoculation method facilitates studies of tar spot and enables an understanding of conditions necessary for the induction of the disease.

This study reveals how little is known about the biology of *P. maydis*. The stromata of *P. maydis* extrude masses of spores onto leaf surfaces under conditions that are unknown; however, spore release appears to occur under environmental conditions also favorable for germination and induction of tar spot [8]. When evaluating the induction of tar spot in the current study, variable results were achieved with a proximity inoculation test. For example, tar spot was induced in the first test (100%), but success was low (20%) in a subsequent test. The inconsistent results may have been due to non-uniform release of spores from the stromata or inconsistent availability of infectious spores in infected leaf pieces. A recent study done under controlled conditions also found that tar spot induction success can vary (50% success) for unknown reasons [17].

Infection was not achieved when using conidia as inoculum under controlled conditions. Although conidia were abundant in extruded spore masses on leaves, they did not germinate and were not infectious under the methodologies used. Nonetheless, we cannot discard the possibility of conidia being infectious under other, unknown conditions. It is hypothesized that conidia of *Phyllachora* species may be spermatia or male gametes involved in sexual reproduction [19], but to date that has not been demonstrated. In the current study, conidia, and ascospores were present in separate stromata 19 dai and 7 days after stromata formed in inoculated plants. This provides insights for understanding the latent period of *P. maydis* but does not clarify the function of conidia. Further research is required to understand the fitness cost or relevance of the conidia in the biology of *P. maydis*.

We developed an efficient approach to collect viable and infectious spores of *P. maydis*. With a vacuum device and syringe tip, contamination of other fungal spores was minimized by targeting individual stromata for spore collection following a similar approach used in rust pathogenicity studies with *Puccinia triticina* and *P. graminis* f. sp. *tritici*, where spores are collected via vacuum from infected *Triticum aestivum* L. (wheat) and used for inoculation [20]. The strategy of using a vacuum collection device or syringe tip like the one used with our method for tar spot has not been used for *Phyllachora* species previously. This is likely because fruiting bodies of *P. maydis* are within stromata [9], and spore extrusion may require stimuli analogous to spores of *Puccinia* spp., which are collected from leaf or stem surfaces of wheat [20]. Our methodology allowed us to collect spores from various sources including green and dried corn leaves. We were able to demonstrate that the spore source and the storage (used immediately vs. -20 ºC for 5 months) are both important aspects for successfully inducing disease. This result also demonstrates how our approach enables fundamental research into the biology of this fungus.

We induced tar spot consistently across four independent experiments and four different corn hybrids. The new method used 20 h in a mist chamber at 19 to 21 ºC, with darkness, 100% RH, and continuous leaf wetness, and later plants were allocated in a greenhouse or growth chamber conditions (Table 4) to allow growth and the development of *P. maydis*. Similarly, in a field study [13], tar spot epidemics were reportedly favored at temperatures between 17 and 23 ºC, % RH > 75%, and > 7 h of nighttime leaf wetness. In previous reports from controlled conditions, induction of tar spot was reported at 20 to 22 ºC, although other conditions were not mentioned [15]; but tar spot was not induced when we evaluated the protocols. Low disease severity (1%) was reported in another study [10] under controlled conditions that included exposing plants to 8 h at 100% RH followed by 5 days at 18 ºC and 12-h photoperiod at 250 µmol/m^2^/s. In the same study, after 5 days at 100% RH, plants were moved to a greenhouse at 18 to 23 °C to allow tar spot development, but % RH, day length, and light intensity were not reported. With the latter protocol, tar spot was not induced in the current study. The new method reported here uses 20 h in the mist chamber, which is less than the 5 days used previously [10], and includes alternating humidifying periods of 10 min every 120 min to achieve continuous leaf wetness without displacing inoculum from leaf surfaces. This period (20 h) is more than the 7 h of leaf wetness reported in field studies [13], but it resulted in tar spot being successfully induced. Nonetheless, the required time of leaf wetness and the mechanisms underlying the etiology of tar spot remain unknown.

In this study, stromata formed 10 to 12 dai in all independent experiments, and ascospores were observed 19 dai (latent period). Previous studies reported periods of 15 [15] to 17 [10] days to stromata formation and incubation periods from 7 to 20 days [10, 15, 17, 18] and they noted chlorosis before stromata appearance. In the current study, chlorosis was not indicative of infection since it was not observed consistently preceding the formation of stromata. We speculate that the lack of symptoms when *P. maydis* infects the plants may be caused by the pathogen’s suppression of host responses, a phenomenon that is typically mediated by cell-death suppression by obligate biotrophic pathogens [21]. Such suppression could also be associated with green symptoms (Fig. 3H3’) that were occasionally observed around stromata on senescing leaves. Moreover, the variable incubation periods reported in this vs. previous studies may be based on different environmental conditions, plant ages, growth stages, and the interaction between different genotypes of the pathogen and host [22–24]. While these studies suggest that infection can occur over a range of corn growth stages, it is unknown how the growth stage of the plants or the pathogenicity and virulence of *P. maydis* influence the latent period and incubation time of tar spot.

The new inoculation method for tar spot reported here is simple to conduct. A challenge, however, is collecting and maintaining field-infected plants as inoculum sources. Although most of our experiments with the new method used spores from stromata on living plants, we also demonstrated that inoculum from frozen leaf samples and from senescing leaves can be used. The discovery that induction of tar spot can be successfully done using spores collected from green leaves that were stored at -20 °C for 5 months markedly expands the utility of this inoculation method. This is a potentially manageable approach to overcome the challenge of maintaining infected living plants in a greenhouse. Compared to living plants, frozen green leaves occupy less storage space and require no maintenance. This finding also suggests fundamental insights into the ability of *P. maydis* spores to survive environmental stressors in ways that may help explain the year-to-year survival of this pathogen.

This study reports a new inoculation method that will be a valuable tool for researching tar spot and *P. maydis* and demonstrates how Koch’s postulates can be completed with this obligate biotroph pathogen. For example, tar spot signs (stromata of *P. maydis*) were found in field-infected corn leaves, spores were isolated from the stroma, the spores induced tar spot after their application to healthy corn plants, and *P. maydis* was extracted from the infected plants. Additionally, the four experiments validated the method’s replicability and consistency by successfully inducing tar spot in 100% of the inoculated plants across greenhouse and growth chamber conditions using different sources of inoculum. The development of this method also provides insights for researching and understanding tar spot.

## Conclusions

Here we describe a new inoculation method to consistently induce tar spot, a significant disease of corn in the Americas. Compared to previously reported protocols, our method is consistent in inducing tar spot and is scalable, robust, and efficient. For example, spores can be collected to produce 50 ml of inoculum, and at least 280 leaves on corn plants at the V2 growth stage can be inoculated in less than 1 h by one person. For scalability, the spore collection step can be repeated to increase the number of collected spores (see methods), facilitating the inoculation of a greater number of plants, as performed for the experiments in the current study. We anticipate that this new method will be useful for breeding for tar spot resistance, understanding the pathogen’s biology, elucidating pathogen-plant interactions, and improving disease management. The method also provides knowledge that can be emulated in the context of other plant diseases.

## Methods

### Plant material

Four corn hybrids (H1 to H4; Table 1) were included in this study. Hybrid H3 was known to be susceptible to tar spot based on field observations in southern Minnesota. Resistance and susceptibility to tar spot were unknown for H1, H2, and H4. Plants were grown by placing four seeds at 2 cm depth in 20 cm diameter plastic pots filled with potting substrate (Sungro Horticulture Professional Growing Mix, Sun Gro Horticulture Distribution Inc., Agawam, MA, U.S.A.) in a greenhouse (22 to 29 °C, 12-h photoperiod with supplemental light from high-pressure sodium lights, and 19 to 76% RH; Supplementary Table 1) located at the Plant Growth Facilities at the University of Minnesota, St. Paul. Temperature and % RH in the greenhouse were monitored constantly using a data logger (VWR Traceable® Excursion-Trac™ USB Datalogging Dual Hygrometer, Radnor Corporate Center, Radnor, PA, U.S.A.). Plants were watered as needed to maintain a moist substrate.

### Pathogen material

#### Field-infected plants

Two living corn plants (VT growth stage) infected with tar spot were collected from a corn production field in southern Minnesota in August 2022. They were placed in pots containing a mixture of field soil (70%) and potting mix (30%) in a greenhouse with conditions as noted above (Supplementary Table 1). The foliage of the plants was washed with water to remove soil and dust. Subsequently, watering occurred at the base of the plants to avoid removing extruded spores from the foliage.

#### Field-infected leaves

Spores of *P. maydis* were also harvested from field-infected (green) and senesced (dried) leaves. Green leaves with signs and symptoms only of tar spot were collected in southern Minnesota in September 2022. The leaves were detached from the stalk, folded, placed in 27 × 27 cm plastic bags (Ziplock®, SC Johnson Golden Rondelle, Racine, WI, U.S.A.), and stored at -20 ºC until use. Another group of green leaves was placed in paper bags, 2 to 3 leaves per bag (AJM Packaging Corporation, Bloomfield Hills, MI, U.S.A.). Dried leaves were also collected in September 2022 to validate our method (experiment 4). Previously, for the preliminary testing of published methods [10, 15], dried leaves were collected from a field in September 2021. Both groups of dried leaves were placed in paper bags and stored at 24 ± 1 ºC until use.

### Inoculum collection and preparation

#### Preliminary experiments

To evaluate the infectious capacity of conidia, conidial masses were collected 18 days after the field-infected plants were moved to a greenhouse as described above using 2 by 2 cm pieces of clear tape (3 M, Scotch® Packaging Tape, St. Paul MN, U.S.A.). The presence of conidia was confirmed by diluting collected masses with 10 µl of double deionized autoclaved water (ddi) and observing with a compound light microscope (Olympus BX41TF, Olympus Corporation, TYO, Japan).

For the proximity inoculation test, a green leaf containing stromata that had been stored at -20 ºC, and was not surface sterilized, was cut transversely using scissors to make 3 cm wide leaf pieces. Each leaf piece was placed onto a 6.7 cm × 5.4 cm cloth pad (American White Cross Laboratories, Inc. New Rochelle, NY, U.S.A.) leaving the adaxial surface exposed for inoculation. For the first test, a leaf piece was prepared 1 day after the leaves were placed in storage; for the second test, 10 leaf pieces were prepared after 17 days of storage.

#### Spore collection – the new vacuum collection method

The presence of ascospores in stromata was assessed before the inoculation day. A subset of stromata (5 to 10) and stromatal extrudates (5 to 10) were manually collected from leaves using a scalpel or forceps, respectively. Each sample’s contents were diluted in deionized water and observed through a compound light microscope to confirm the presence of ascospores.

Collection of spores from field-infected plants occurred in the greenhouse under the conditions mentioned for the above pathogen material at 27, 54, and 57 days after plants were introduced to the greenhouse. To collect spores, a plastic tip cut from a 3 ml syringe (Monoject ™ Syringes; Dublin, OH, U.S.A.) was inserted into a plastic elbow connector, which was connected to the plastic tube attached to the canister of the vacuum (The elbow connector, plastic tube, and the canister are accessories of the vacuum: Heavy Duty Suction Machine #18,600; Medical Depot, Inc. dba Drive DeVilbiss Healthcare, Port Washington, NY, U.S.A.) (Supplementary Fig. 1). The syringe tip was used to collect visible masses of spores (orange, yellow, red, and white) from the surface of individual stroma while the vacuum was set to its highest power level. To collect spores from inside the stromata, the syringe tip was pressed flush over the stromata and rubbed to disrupt the stromata and create a vacuum seal (40 to 60 kPa). After repeating this process with 300 to 400 stromata, a powdery material of variable color (orange to white) was visible in the collection canister. To collect spores that became trapped in the tubing, 2 to 5 ml of 0.01% Tween 20 were drawn through the syringe tip and collection tube until the liquid was deposited in the canister. Subsequently, the contents of the collection canister were transferred to 50 ml tubes.

Spores were also collected from frozen green leaves. Non-surface sterilized leaves were placed with the adaxial surface facing up on a lab bench cleaned with 70% ethanol and allowed to thaw for 5 min until spore collection. On a different bench, a field-infected senesced leaf that had been stored at 24 ± 1 °C for 5 months was surface sterilized in 0.5% sodium hypochlorite for 30 s, followed by 30 s in 95% ethanol [9], cut into irregular pieces, and then, immersed in ddi water for 5 min to soften stromata and facilitate spore collection. Spores were collected from 40 to 80 stroma with a new collection device constructed similarly to devices used to collect spores of *Puccinia* spp. but with a flexible collector tube (Bic Cristal Original Ink Chamber, Bic Inc., Shelton, CT, U.S.A.) to facilitate the manipulation of stromata and spore collection (Supplementary Fig. 1). During collection, 20 µl of 0.01% Tween 20 were applied to each stroma to aid with the flow of spores through the device’s collection tube resulting in a dark-colored liquid in the deposit tube (Olympus Plastics Cat. 22–281, 1.7 ml Microtubes, Clear Polypropylene, Genesee Scientific, El Cajon, CA, U.S.A.). Spores were collected from green and dried leaves separately, the device was sterilized with 70% ethanol between collections, and the vacuum tube was replaced between each collection to avoid mixing spores from different sources. Spores from each source were transferred separately to 50 ml tubes.

Ascospore concentration was adjusted to 10^4^ to 10^5^ per ml of 0.01% Tween 20 with the aid of a hemocytometer (Bright-Line Hemacytometer Cat. 3100; Hausser Scientific, Horsham, PA, U.S.A.) and a compound light microscope. The inoculum contained ascospores and conidia. Spores collected from field-infected plants were used within 1 h after collection and spores collected from detached leaves were used within 2 h after collection. The volume of spore suspension was prepared based on the number of plants to inoculate in each experiment. For example, experiments 1 to 3 (3 to 4 leaves inoculated per plant and up to 72 plants; Table 2) each required 50 ml of inoculum; and experiment 4 (1 leaf inoculated per plant and up to 28 plants; Table 2) required 5 ml of inoculum from green leaves and 5 ml from dried leaves.

### Inoculation

#### Preliminary experiments

Inoculation occurred in a greenhouse with the conditions described above. In the initial test, conidial masses adhering to a 2 by 2 cm piece of clear plastic packaging tape were placed onto the adaxial surface of healthy-appearing green leaves of 16 plants (V3 growth stage). No application occurred in the control group (16 plants). After inoculation, 8 of the 16 inoculated plants and 8 of the non-inoculated plants were placed in the greenhouse and the rest of the plants were placed in a mist chamber (0.8 m × 1.4 m × 1 m) equipped with a humidifier (VICKS Ultrasonic Cool Mist Humidifier Model v5100-n; Kaz, Inc. NY, U.S.A.) that cycled 10 min every 120 min to maintain RH at 100% for 48 h. The misting cycles were implemented using a controller (Trident T3A-1 Zone; Phytotronics Inc., Earth City, MO, U.S.A.). Temperatures in the mist chamber were between 20 and 25 °C in the dark. After 48 h in the mist chamber, the plants were moved to a greenhouse (19 to 22 °C with a 12-h photoperiod) and placed with the other 16 plants in a completely randomized design. Each plant was considered a replicate. Plants were then monitored daily for 30 days to assess the development of symptoms and signs.

Plants were inoculated for the proximity test on a lab bench. Each cloth pad containing a piece of field-infected corn leaf was folded over healthy-appearing green leaves of V3 growth stage plants to cover both surfaces. The leaf piece was secured to the plants by taping the cloth pad to the leaves, and 3 ml of ddi water were applied to favor spore release and germination. Subsequently, the plants were placed in a growth chamber set to 19 °C, 85 to 100% RH, and 8-h photoperiod and arranged in a completely randomized design. The cloth pads and leaf pieces were removed 3 dai and the plants were monitored daily for 30 days to assess symptoms and signs.

#### Inoculation – the new method

Inoculation was conducted on plants with healthy-appearing green leaves at the V2 growth stage. Prior to inoculation, a humidifier increased the RH to 100% in the mist chamber. Leaves were inoculated by spreading the spore suspension (inoculum) on the entire surface of 3 to 4 leaves (experiments 1 to 3) and 1 leaf (experiment 4) per plant using a flat 1.3 cm paint brush (Plaid Enterprises, Inc., Norcross, GA, U.S.A.) (Fig. 2C). The inoculum was used within 1 (experiments 1 to 3) to 2 (experiment 4) h after collection. After all four plants in pots were inoculated, the pots were immediately placed in the chamber to avoid desiccation of the inoculum. During inoculation, the humidifier was set to run at half mist intensity to avoid water accumulation on the leaves and displacement of the inoculum due to free water on the leaf surfaces. When inoculations were completed and all plants were moved to the mist chamber, the humidifier was set to run at high mist intensity for 10 min cycles, every 120 min for 20 h in the dark to maintain 100% RH and leaf wetness (Table 3). Temperature and % RH in the mist chamber were recorded with a data logger (Temp. RH Baro. USB Data Logger 88,163; AZ, No. 3 − 2, Jianguo Rd., Taichung City, 427 Taiwan R.O.C). After 20 h in the mist chamber, plants were either moved to a greenhouse (20 to 25 °C, 12-h photoperiod with light intensity 97 to 536 µmol/m^2^/s, and 19 to 62% RH) or to a growth chamber (19 to 20 ºC, 29 to 48% RH and 8-h photoperiod with light intensity 131 to 320 µmol/m^2^/s) (Table 4). In the greenhouse and growth chamber, temperature, and % RH were monitored using a data logger as described above (plant material section), and light intensity was measured using a light meter (Apogee ePAR Meter MQ-500, Apogee Instruments, Inc., North Logan, UT, U.S.A.).

### Symptom development and assessment following inoculation using the new method

Tar spot induction was confirmed when stromata of *P. maydis* appeared on the foliage of inoculated plants. Plants were visually assessed daily to observe and record symptoms and signs. The day when stromata were first observed was recorded and stromata were counted and leaves were photographed at 26 dai (experiment 1) and at 19 dai (experiments 2, 3, and 4) (Fig. 3). To assess spore type in stromata on inoculated plants, a random subset of 5 stromata from each experiment were collected, cut, and the contents were observed using a compound light microscope. Leaves containing stromata were stored at -20 °C.

### A step-by-step description of the new method

- To obtain inoculum from live plants, corn plants infected with *P. maydis* can be collected from the field and maintained in pots in a greenhouse or plants can be inoculated and maintained in a greenhouse (20 to 25 ºC, 12-to-8-h photoperiod with light intensity 97 to 536 µmol/m^2^/s, and 19 to 62% RH) (Table 4). Alternatively, infected green leaves can be collected from the field, placed in plastic bags (2 to 3 leaves per bag), and stored at -20 ºC. This is a critical step to plan in advance to have sufficient stromata for spore collection and inoculation.
- Before spore collection, confirm the presence of ascospores in stromata or stromatal exudates using a compound light microscope. Extruded spore masses are collected from the stromata using forceps, or stromata are dissected from the leaves to assess their contents. This step can occur before the inoculation day.
- After the presence of ascospores is confirmed, prepare a vacuum collection device to collect ascospores (Supplementary Fig. 1). Spores can be effectively collected at 40 to 60 kPa. To obtain 50 ml of inoculum containing 10^4^ to 10^5^ ascospores per ml from field-infected plants, collect spores from 300 to 400 stromata and ensure that powdery material is deposited in the vacuum’s canister. The number of stromata and leaves to target for collection will vary depending on spore availability and the number of plants and leaves to be inoculated.
- Following the collection of spores from stromata, remove spores retained in the tubing by suctioning 2 to 5 ml of 0.01% Tween 20 through the spore collector to deposit spores into the canister. The volume to intake will vary depending on the instrument used for the collection.
- Gently stir the diluted ascospore suspension to keep spores suspended and determine ascospore concertation using a hemocytometer and compound light microscope. Adjust ascospore concentration with 0.01% Tween 20 to 10^4^ to 10^5^ ascospores per ml. Conidia will typically be present and outnumber ascospores.
- Before plants are inoculated, set a humidifier to increase RH to 100% in a dark mist chamber set to 19 to 21 ºC and 100% RH (Table 3).
- Inoculate leaves within 2 h after ascospore collection by applying the spore suspension to the entire adaxial surface of corn leaves using a 1.3 cm flat brush. Place inoculated plants into the mist chamber immediately after inoculation to avoid desiccation of the inoculum.
- When all plants are inoculated and inside the mist chamber, set a humidifier to run cycles of 10 min every 120 min to maintain 100% RH for 20 h in darkness.
- After 20 h in the mist chamber, move the plants to a greenhouse or growth chamber maintained at 19 to 25 ºC, 8 to 12-h photoperiod, light intensity 97 to 536 µmol/m^2^/s, and 19 to 62% RH (see temperature, %RH and light intensity ranges in Table 4).
- Add water and fertilizer to soil in pots as needed to maintain the health of corn plants. Check for symptoms and signs of tar spot daily. Symptoms of disease development can sometimes be first observed as chlorotic flecking on the leaf cuticle where stromata emerge (Figs. 2 and 3).

### Data analysis

Data from each of the four experiments were processed and analyzed individually through the R software v.4.2.2 [25] and RStudio v.2022.12.0 + 35 [26]. The counts of stromata per leaf were not a continuous variable and did not meet the requirements of homoscedasticity for analysis of variance. The data were subjected to Tukey’s Ladder of Power transformation with the function ‘transformTukey’ within the R package ‘rcompanion’ v. 2.4.21 [27]. Subsequently, the effects of hybrid, location (greenhouse or growth chamber), and the number of stromata per inoculated leaf among different hybrids were assessed through analysis of variance (ANOVA). The data were fitted to a linear model ‘lm’, and then analyzed with ANOVA using the ‘aov’ function. After ANOVA, the normality of the residuals was evaluated through a Kolmogorov-Smirnov test with ‘ks.test’ to determine if data could be analyzed using Tukey’s Honestly Significant Difference test to determine differences among hybrids, locations, and inoculated leaves (Supplementary Tables 2 to 6). Figures were produced through the suite of packages within ‘tidyverse’ v.1.3.2 [28].

### Electronic supplementary material

Below is the link to the electronic supplementary material.

**Supplementary Figure 1:** Depiction of the vacuum collection device and syringe tip used for collection of *Phyllachora maydis* spores from corn leaves prior to inoculation of plants with the new method. **Supplementary Figure 2:** *Phyllachora maydis* ascospores germinating 30 min after dilution in 0.01% Tween20 at 25 ± 1 ºC. Ascospores were collected from stromata in field-infected plants using a vacuum and syringe tip. **Supplementary Table 1:** Conditions in a greenhouse during the development of *Phyllachora maydis* on field-infected corn plants. **Supplementary Table 2:** Analysis of variance (ANOVA) for experiment 1. **Supplementary Table 3:** Analysis of variance (ANOVA) for experiment 2. **Supplementary Table 4:** Analysis of variance (ANOVA) for experiment 3. **Supplementary Table 5:** Analysis of variance (ANOVA) for experiment 4. **Supplementary Table 6:** Analysis of variance (ANOVA) for all experiments.

## Data Availability

For reproducibility, the R code used for data analysis is available at: https://github.com/jsolorzano734/tarspot_corn.

## Abbreviations

H: Hybrid
% RH: Percentage relative humidity
U.S.: United States of America
V(n): Vegetative growth stage
ddi: Double deionized autoclaved water
dai: Days after inoculation
Green leaves: Field-infected green leaves
Dried leaves: Field-infected senesced leaves
