# Supplementary material for: A new and effective method to induce infection of Phyllachora maydis into corn for tar spot studies in controlled environments
Source: Plant Methods. 2023 Aug 11;19:83. doi: 10.1186/s13007-023-01052-8 (PMC10416423; doi:10.1186/s13007-023-01052-8)
Supplement: Supplementary file 1 — Supplementary Figure 1: Depiction of the vacuum collection device and syringe tip used for collection of Phyllachora maydis spores from corn leaves prior to inoculation of plants with the new method. Supplementary Figure 2: Phyllachora maydis ascospores germinating 30 min after dilution in 0.01% Tween20 at 25 ± 1 ºC. Ascospores were collected from stromata in field-infected plants using a vacuum and syringe tip. Supplementary Table 1: Conditions in a greenhouse during the development of Phyllachora maydis on field-infected corn plants. Supplementary Table 2: Analysis of variance (ANOVA) for experiment 1. Supplementary Table 3: Analysis of variance (ANOVA) for experiment 2. Supplementary Table 4: Analysis of variance (ANOVA) for experiment 3. Supplementary Table 5: Analysis of variance (ANOVA) for experiment 4. Supplementary Table 6: Analysis of variance (ANOVA) for all experiments. [file 13007_2023_1052_MOESM1_ESM.docx]

**A new and effective method to induce infection of *Phyllachora maydis* into corn for tar spot studies in controlled environments**

José E. Solórzano^1*^, Shea E. Issendorf^1^, Milton T. Drott^1,2^, Jill C. Check^3^, Emily M. Roggenkamp^3^, C. D. Cruz^4^, Nathan M. Kleczewski^5^, Carlos C. Gongóra-Canul^4,6^, and Dean K. Malvick^1*^

**Institutional addresses**

^1^ University of Minnesota, Department of Plant Pathology, St. Paul, MN 55108, U.S.A.

^2^ United States Department of Agriculture-Agricultural Research Service, Cereal Disease Laboratory, St. Paul, MN, 55108, U.S.A.

^3^ Michigan State University, Department of Plant, Soil and Microbial Sciences, East Lansing, MI 48824, U.S.A.

^4^ Purdue University, Department of Botany and Plant Pathology, West Lafayette, IN 47907, U.S.A.

^5^ GROWMARK Agronomy Services, Bloomington IL, 61702, U.S.A.

^6^ Tecnológico Nacional de México, Instituto Tecnológico de Conkal, YU 97345, Mexico

***Corresponding authors:**

J. E. Solórzano; E-mail: [ortiz432@umn.edu](mailto:ortiz432@umn.edu)

D. K. Malvick; E-mail: [dmalvick@umn.edu](mailto:dmalvick@umn.edu)

**SUPPLEMENTARY MATERIAL**


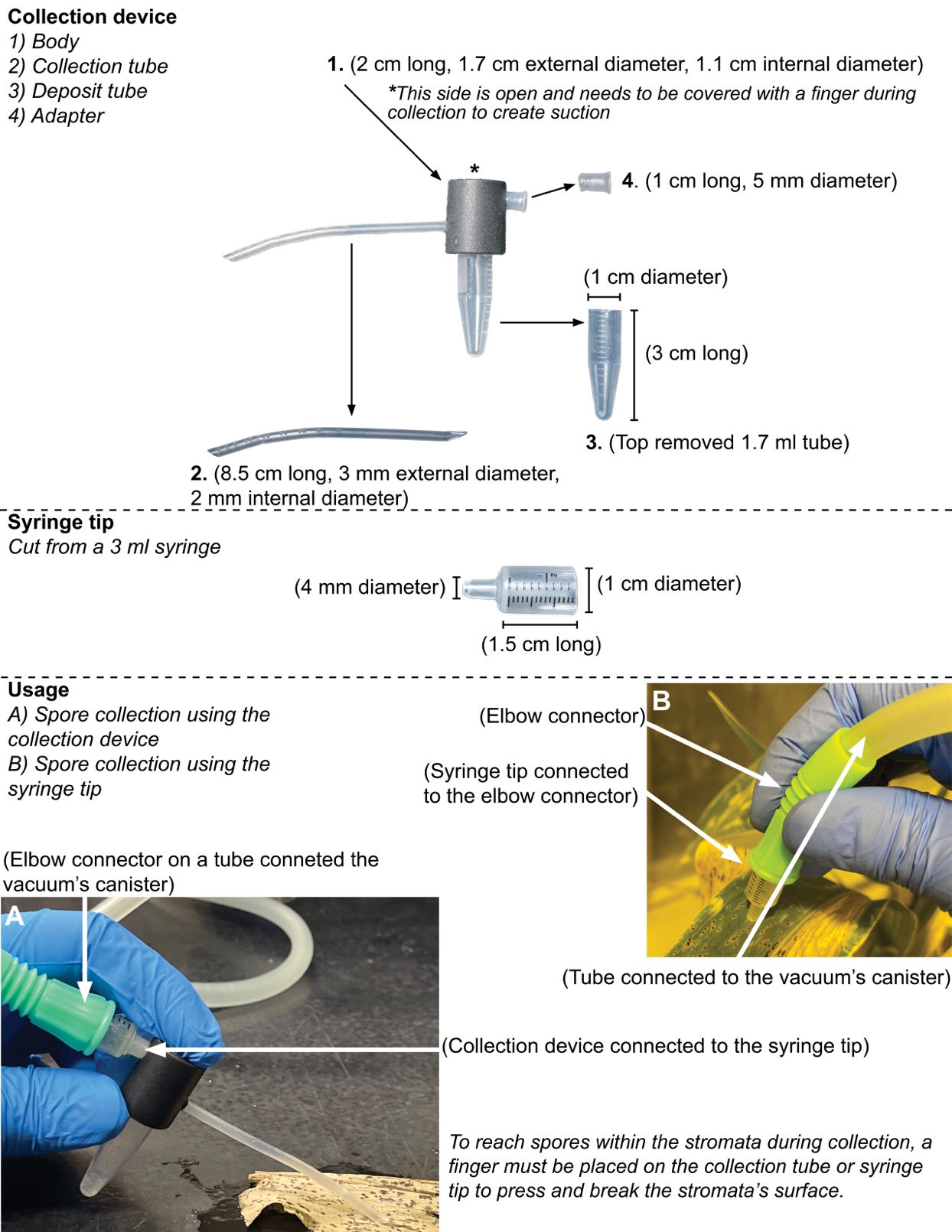


**Supplementary Figure 1.** Depiction of the vacuum collection device and syringe tip used for collection of *Phyllachora maydis* spores from corn leaves prior to inoculation of plants with the new method.


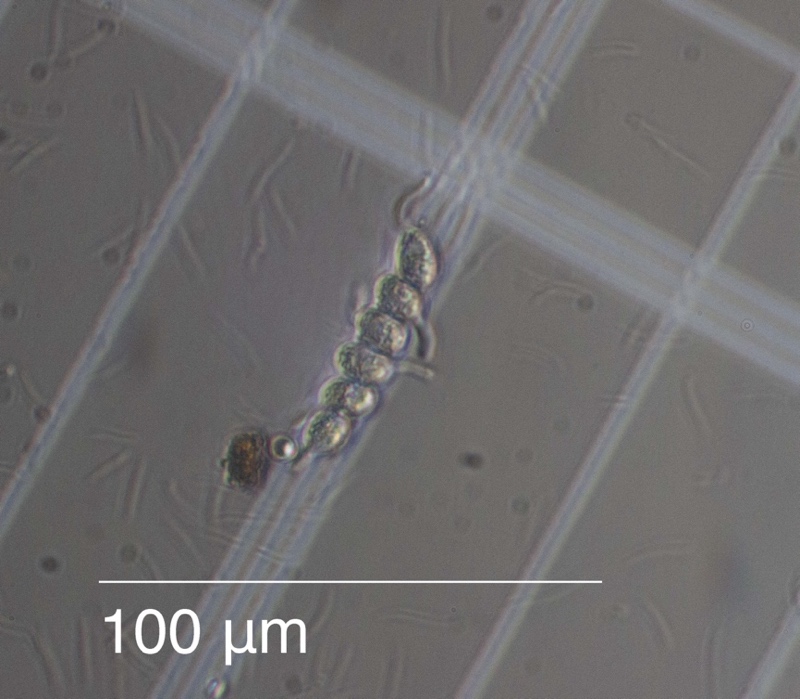


**Supplementary Figure 2.** *Phyllachora maydis* ascospores germinating 30 min after dilution in 0.01% Tween20 at 25 ± 1 ºC. Ascospores were collected from stromata in field-infected plants using a vacuum and syringe tip.

**Supplementary Table 1.** Conditions in a greenhouse during the development of *Phyllachora maydis* on field-infected corn plants.

| Range | % RH § | Temperature ºC § |
| --- | --- | --- |
| Minimum | 19 | 22 |
| Mean | 48 | 24 |
| Maximum | 76 | 29 |

§ Data were collected for 57 days, starting the day when field-infected plants were introduced to the greenhouse to the last spore collection.

**Supplementary Table 2.** Analysis of variance (ANOVA) for experiment 1.

|  | **Df** | **Sum Sq** | **Mean Sq** | **F-value** | **Pr( > F)** |
| --- | --- | --- | --- | --- | --- |
| Leaves | 3 | 0.00581 | 0.001937 | 0.326 | 0.807 |
| Locations | 1 | 0.00972 | 0.009716 | 1.633 | 0.212 |
| Residuals | 27 | 0.16067 | 0.005951 |  |  |

ANOVA was conducted for the number of stromata that developed on inoculated leaves (3 to 4) of hybrid H1 (Table 1) in greenhouse and growth chamber conditions (locations) following inoculation with *P. maydis* (Fig. 4A). The control group was not included since tar spot was not induced in it. Significance codes: *P* ≤ 0.001 ‘***’, *P* ≤ 0.01 ‘**’, *P* ≤ 0.05 ‘*’, *P* ≤ 0.1 ‘.’.

**Supplementary Table 3.** Analysis of variance (ANOVA) for experiment 2.

|  | **Df** | **Sum Sq** | **Mean Sq** | **F-value** | **Pr( > F)** |  |
| --- | --- | --- | --- | --- | --- | --- |
| Leaves | 2 | 0.0159 | 0.00793 | 0.757 | 0.471 |  |
| Hybrid per location | 6 | 0.7035 | 0.11725 | 11.196 | 1.09E-09 | *** |
| Residuals | 108 | 1.1311 | 0.01047 |  |  |  |

ANOVA was conducted for the number of stromata that developed on inoculated leaves and the number of stromata between four corn hybrids (H1 to H4; Table 1) in greenhouse vs. growth chamber conditions (location) (Fig. 4B). The control group was not included since tar spot was not induced in it. Significance code: *P* ≤ 0.001 ‘***’, *P* ≤ 0.01 ‘**’, *P* ≤ 0.05 ‘*’, *P* ≤ 0.1 ‘.’.

**Supplementary Table 4.** Analysis of variance (ANOVA) for experiment 3.

|  | **Df** | **Sum Sq** | **Mean Sq** | **F-value** | **Pr( > F)** |  |
| --- | --- | --- | --- | --- | --- | --- |
| Leaves | 3 | 0.0364 | 0.012138 | 1.791 | 0.15439 |  |
| Hybrids | 3 | 0.0905 | 0.030165 | 4.451 | 0.00576 | ** |
| Residuals | 92 | 0.6235 | 0.006777 |  |  |  |

ANOVA was conducted for the number of stromata that developed on inoculated leaves (3 to 4) and the number of stromata between four corn hybrids (H1 to H4; Table 1) (Fig. 4C). The control group was not included since tar spot was not induced in it. Significance codes: *P* ≤ 0.001 ‘***’, *P* ≤ 0.01 ‘**’, *P* ≤ 0.05 ‘*’, *P* ≤ 0.1 ‘.’.

**Supplementary Table 5.** Analysis of variance (ANOVA) for experiment 4.

|  | **Df** | **Sum Sq** | **Mean Sq** | **F-value** | **Pr( > F)** |
| --- | --- | --- | --- | --- | --- |
| Hybrids | 3 | 0.0629 | 0.02097 | 0.841 | 0.486 |
| Residuals | 23 | 0.5738 | 0.02495 |  |  |

ANOVA was conducted for the number of stromata that developed across four corn hybrids (H1 to H4; Table 1) following inoculation with *P. maydis* (Fig. 4D). The control group was not included since tar spot was not induced in it. Significance codes: *P* ≤ 0.001 ‘***’, *P* ≤ 0.01 ‘**’, *P* ≤ 0.05 ‘*’, *P* ≤ 0.1 ‘.’.

**Supplementary Table 6.** Analysis of variance (ANOVA) for all experiments.

|  | **Df** | **Sum Sq** | **Mean Sq** | **F-value** | **Pr( > F)** |  |
| --- | --- | --- | --- | --- | --- | --- |
| Experiment-location | 5 | 0.2831 | 0.05662 | 15.872 | 1.11E-13 | *** |
| Leaves | 3 | 0.0428 | 0.01427 | 3.999 | 0.00826 | ** |
| Residuals | 266 | 0.9488 | 0.00357 |  |  |  |

ANOVA was conducted for the number of stromata per all experiments in the greenhouse and growth chamber (experiment-location) and the number of stromata on inoculated leaves (1 to 4) of four corn hybrids (H1 to H4; Table 1) following inoculation with *P. maydis*. The control group was not included since tar spot was not induced in it. Significance codes: *P* ≤ 0.001 ‘***’, *P* ≤ 0.01 ‘**’, *P* ≤ 0.05 ‘*’, *P* ≤ 0.1 ‘.’.
